# Building trust during crises by engaging with existing distrust

**DOI:** 10.3389/fpubh.2025.1570243

**Published:** 2025-05-02

**Authors:** Tarun Kattumana, Heidi J. Larson

**Affiliations:** ^1^Husserl Archives and Center for Phenomenology and Continental Philosophy, Institute for Philosophy, KU Leuven, Leuven, Belgium; ^2^Access-To-Medicines Research Centre, KU Leuven, Leuven, Belgium; ^3^Department of Infectious Disease Dynamics, London School of Hygiene & Tropical Medicine, London, United Kingdom; ^4^Institute for Health Metrics & Evaluation, University of Washington, Seattle, WA, United States

**Keywords:** trust, distrust, crisis, COVID-19 pandemic, vaccine hesitancy, medical populism

## Abstract

It has often been argued that trust cannot be built during crises. While this line of thinking recognizes the importance of building trust before or after crises, it negates the possibility of building trust during a crisis. This paper offers two scenarios to argue for the possibilities of building trust during crises by engaging with existing distrust, with special reference to examples during the COVID-19 pandemic. The first considers distrust towards vaccines and public health interventions among Black Americans that was addressed through interventions that partnered with trusted third parties during the COVID-19 pandemic. The second discusses the Indian transgender community where trust networks built between public health organizations and activists groups was mobilized to engage with existing distrust during the COVID-19 pandemic. Despite the success of these interventions, this perspective paper ends by clarifying that engaging with distrust does not have to result in achieving public health goals with a brief considerations of two cases: the polio vaccine boycott in Nigeria (2003) and medical populism in the United States during the COVID-19 pandemic. These examples highlight that if existing distrust toward the medical system is not addressed, then space is created during crises-periods for distrust to be mobilized in ways that compromise public health goals.

## 1 Trust, distrust, and crises

Trust can be defined, following Whyte and Crease, as “deferring with comfort and confidence to others, about something beyond our knowledge or power, in ways that can potentially hurt us” [(1), p. 412]. One party trusts another with an issue or concern that is beyond their control. As a consequence, the trusting party is dependent on the goodwill of the trusted; a dependence that can be abused. What this highlights is that trust does not come with guarantees and it is always possible that the trusting party can be hurt or let down by the trusted. When trusting, the possibility of being hurt is taken on board in the hope that those we trust will not respond with ill-will. In other words, trust foregrounds vulnerability on part of the trusting party and good will on part of the trusted.

The vulnerabilities of trust are accepted in varying degrees because it reduces complexity. For example, when driving decisions have to be made quickly without recourse to time consuming reflection or discussion with other drivers. It is always possible, for instance, that we could be injured by those who drive irresponsibly or vehicles that have not passed adequate safety regulations. Despite this, there is a general tendency to trust other drivers to co-operate to varying extents to reach the intended destination. Without trust road travel would be impossible. Put simply, trust allows for the “reduction of complexity” which in turn enables pursuing goals that would otherwise be impossible [(2), p. 25]. An additional caveat at play in the driving example is an impersonal “system trust” that the rules and regulations of the traffic system will continue to function as expected [(3), p. 432]. In sum, trust can be defined as a “relationship that exists between individuals, as well as between individuals and a system, in which one party accepts a vulnerable position, assuming the best interests and competence of the other, in exchange for a reduction in decision complexity” [(4), p. 1599].

This is not to claim that all forms of trust are reflective leaps of faith for the sake of reduced complexity. Trust is also the result of routines or what could be termed habitual trust (5). Most drivers do not ponder endlessly on whether they can trust the conduct of other drivers before crossing a signal. There is a habitual trust among most drivers that others can be trusted not to break traffic rules and crash into them. However, the qualifier ‘most’ indicates a crucial dimension: it is not necessary that everyone exhibit trust all the time. Distrust remains a potential response but one that is closely intertwined with trust. Consider Steven Shapin’s ‘skeptical experiment’ concerning processes by which the factual claim ‘DNA contains cytosine’ is verified [(6), pp. 17–19]. Among the many details of the experiment is the precipitate of certain chemical reactions that is taken to be DNA, a conclusion that Shapin notes would not be questioned by most participants in laboratory activities. Such taken-for-granted acceptance might be cause for skepticism and result in subjecting the precipitate to additional tests. This would be time-consuming, cost money, and disrupt work flow. But such distrust is reasonable. Shapin’s point, however, is that distrust still requires trust in the competence of those who prepared the extra tests or the efficacy of the tests themselves. It highlights that acts of distrust require some form of trust that is always already operative and implicitly factored in [(6), p. 19]. This form of habitual trust is taken for granted and not even recognized as a form of trust. For Annette Baier, this is because we function within “a climate of trust” that we “inhabit [like] an atmosphere and notice it as we notice air, only when it becomes scarce or polluted” [(7), p. 607].

Discussion of trust and distrust highlights another important feature: the expectational dimension. Following Niklas Luhmann, trust functions as a “generalization” that extrapolates from one state of affairs to form a series of expectations that can be extended to others [(2), p. 26]. More precisely, trust represents a positive set of expectations that leads to a generalization that similar agreeable circumstances can be expected in the future. Distrust, on the other hand, is not merely the lack of trust but a “functional equivalent” and a series of expectations in its own right [(2), p. 71]. The caveat being that while trust represents positive expectations, distrust refers to negative expectations. Furthermore, like trust, distrust also reduces complexity as the expectation of undesirable outcomes leads to protective or preemptive action [(2), p. 72]. But, as Shapin’s ‘skeptical experiment’ shows, trust and distrust are not mutually exclusive but coexist in a myriad of multifaceted and richly textured ways [(8), pp. 442, 450]. For example, most organizations have institutional check mechanisms that function on the basis of distrust based negative expectations that seek to preemptively limit violations thereby creating an environment of trust.

Given the above nuances, there is a complicated relationship between trust, distrust, and complex social crises. Consider the COVID-19 pandemic whose impact was not limited to the health sector but pervaded multiple social sectors (9, 10). In other words, the pandemic constituted a polycrisis where the overall impact of deeply intertwined and simultaneously occurring crises far exceeds the consequences of any single crisis (11). This results in a heightened increase in complexity that would seem to require trust and its ability to reduce complexity. But the manner in which complex social crises play out would initially seem to limit such prospects. Staying with the pandemic, public health interventions to control the spread of SARS-CoV-2 disrupted regular routines and familiar practices destabilizing the bedrock of habitual trust that permeates everyday life [(12–14), pp. 219–222]. The loss of habitual trust is further accentuated by disruptions to systemic trust. For instance, the public transport system or school system in various countries were discontinued or altered significantly such that they could not function as expected. For these reasons, complex social crises are not expected to set up trust based positive expectations of agreeable circumstances playing out in the future and instead raises the prospect of distrust based negative expectations. This has contributed to the understanding that building trust has better prospects before a crisis rather than during a crisis. Or as Scott Keller argues “You cannot build trust when a crisis happens. You build trust before a crisis happens” (15). This perspective paper challenges the strong opposition drawn between the likelihood of building trust before crises to its seeming impossibility during crises. By discussing concrete cases during the COVID-19 pandemic, this paper argues that trust can be built during crises, albeit indirectly, by engaging existing distrust in a manner that leverages previously trusted relationships.

## 2 Black Americans, distrust, and vaccine hesitancy

During the early phase of the COVID-19 pandemic, vaccine hesitancy was reported among Black Americans (16, 17). Contributing factors included concerns over vaccine safety and distrust of the public health system [(18), pp. 649–650, (19)]. There were also reports of Black Americans lacking access to vaccination centers, protective gear, and testing equipment while also receiving inadequate care or being sent home despite showing symptoms (20). A prominent case being Dr. Susan Moore, a family physician from Indianapolis, who was hospitalized with COVID-19 in November 2020 (21). In a self-recorded Facebook video post at the hospital, Dr. Moore recounted how she had to beg the medical staff for adequate treatment. Despite being a doctor who could express her need for pain medication in the appropriate medical terms, she received racially biased treatment; an occurrence that has been shown to arise owing to false beliefs regarding disparities in pain perception between Black and White Americans (22). Dr. Moore’s video ended with the haunting statement that ‘This is how Black people get killed–when you send them home and they do not know how to fight for themselves’ (23). Within a day of being discharged Dr. Moore was hypotensive and needed to go to another hospital. However it was too late, Dr. Moore’s health deteriorated and she passed away at the age of 52.

Dr. Moore’s video was widely shared and speaks to a generalized distrust of the medical system among Black Americans. Put differently, there is a series of negative expectations among sections of the Black American community regarding how they will be received and treated by the medical establishment. Distrust among Black Americans has a historical dimension that can be traced back to the Antebellum period and continues into the 20th century with prominent examples like the Tuskegee Syphilis Experiment (24). When using historical examples, the aim is not to overemphasize the weight of a single event but gesture to the manner in which the healthcare system treated Black Americans leading to a generalized distrust that is experienced in different degrees and intensities by members of this group (25).

Against this backdrop of institutional racism and distrust based negative expectations, the reluctance to vaccinate represents a preemptive and protective gesture of not wanting to take a chance with, or be at the behest of, the health system. However, as the pandemic unfolded there was a rapid increase in the intention to vaccinate among Black Americans [(26), pp. 7–8, (27)]. This increase has been attributed to proactive efforts that directly address existing distrust in a manner that acknowledges Black American needs and concerns. For example, the Black Coalition Against COVID in Washington D.C collaborated with four Black medical schools and a number of medical organizations trusted by the Black American community to organize virtual town halls (28). Through these events and others, organizers spoke to existing fears and concerns that drive distrust while sharing information about the safety and effectiveness of the vaccine (29). Similarly, the Black Doctors COVID-19 Consortium in Philadelphia directly engaged with Black Americans by collaborating with trusted partners, organizing free testing services in churches and community centers, and provided targeted information about COVID-19 vaccines. These efforts proactively addressed existing dissatisfaction with inadequate access to testing that has been identified as one of the reasons why SARS-CoV-2 has disproportionately affected Black Americans (30). Building on these initiatives the Consortium could set up positive expectations that contributed to a willingness to vaccinate (31).

## 3 Indian transgender community, distrust, and vaccine hesitancy

The Indian transgender community experiences discrimination and exclusion. Transgender persons tend to leave home or be forced out by family members [(32), p. 9]. This in turn leads to having inadequate documentation to get jobs, own property, or avail of state welfare schemes many of which require valid identification [(33), pp. 8–9]. As a result, members of this community are forced to beg, take up sex-work, or perform culturally assigned roles (blessing babies or dancing during ceremonies) for their livelihoods [(32), pp. 4–9]. Although there have been improvements in the legal domain with the Government of India’s NALSA verdict in 2014, which mandated the addition of a “third gender” column in all government documents as a sign of legal recognition, much remains to be done to address existing patterns of socio-cultural discrimination and exclusion. The COVID-19 pandemic only served to exacerbate the intersectional brunt of transgender persons (34). Stay-at-home orders, the closure of public spaces, and discontinued public transport to curb the spread of SARS-CoV-2 made the prospect of making a living through begging, sex-work, or partaking in culturally assigned roles mentioned above exceedingly difficult.

These socio-economic and cultural realities lead transgender persons to have distrust based negative expectations of how they will be treated in public; expectations that extend to the medical system as well. Members of the transgender community are subject to conscious misgendering, physical/verbal abuse, and face the prospect of being denied access to HIV testing, sexual health services, and antiretroviral treatment [(35), pp. 12–13]. All of these factors contribute to an overall strained relationship with medical institutions that in turn impacts vaccine uptake [(36), pp. 6]. During the COVID-19 pandemic, the official registration form for the vaccine only had the option of ‘others’ as opposed to culturally preferred categories for those not wanting to choose male or female (37). Furthermore, there was a lack of clarity as to how the vaccine would interact with existing medication for HIV/AIDS or Hormone Replacement Therapy (HRT) [(38), p. 8]. All these factors contributed to an indecisiveness associated with vaccine hesitancy. The COVID-19 vaccine uptake among transgender persons in India was extremely low at 3.97 percent during the devastating second wave of the pandemic (39).

Successful initiatives to improve vaccine uptake directly addressed existing distrust while collaborating with third parties already trusted by the transgender community. Consider the Momentum Routine Immunization Transformation and Equity Project and their efforts to address the lack of clarity regarding how the vaccine would interact with medication for HRT or HIV/AIDS in Chandigarh, India (38). The project worked together with the ‘Third Gender Welfare Board’ set up by the state government along with transgender activists and those holding leadership positions within the transgender community. While the project does not provide data specific to transgender persons in Chandigarh, collaborating with third-parties who were trusted by the community contributed to administering 6.1 million vaccine doses to marginalized communities in India. Similarly Swasti, an organization based in Bangalore, have published a COVID-19 Vaccination Playbook that emphasizes that it is “imperative to consult with community representatives—at every step, at every phase” (40). Vaccination camps set up on the basis of collaboration with representatives from the transgender community who have trained personnel to be knowledgeable about concerns of specific to this group, were a crucial trust building effort in the context of the COVID-19 pandemic. These initiatives have resulted in close to 6,206,000 people being vaccinated across 24 states and union territories in India including transgender men and women, sex workers, and informal workers among other vulnerable groups (41).

## 4 Conclusion: cautionary cases and the need to engage with existing distrust

Both trust and distrust are complex constructs that can be mobilized in multiple ways. Examples of Black Americans and the Indian Transgender community show that efficaciously engaging distrust can set the stage to build trust required for achieving public health goals. But this does not have to be the case. There are other instances of mobilizing distrust that compromised public health outcomes. One prominent example is what Gideon Lasco and Nicole Curato term “medical populism,” or a “political style that constructs antagonistic relations between ‘the people’ whose lives have been put at risk by ‘the establishment’” [(42), p. 1]. In the present context, the establishment refers to institutions, actors, and networks that are broadly associated with public health practice.

An example of medical populism before the pandemic is the polio vaccine boycott (2003) in Kano State, Northern Nigeria. This boycott built on long standing suspicion towards Western medicine that arose due in part to problematic encounters with the pharmaceutical industry. In 1996, families in Kano State raised concerns with Pfizer for setting up trials for an experimental meningitis drug without adequately addressing the risks for participants [(43), p. 337]. These experiences combined with existing fears that the polio vaccine contained anti-fertility drugs and was intended to control the growth of the Muslim population also contributed to the boycott (44). However, the crucial node was the mobilizing of distrust by local politicians intending to resist the federal government in the context of religious and political tensions in Nigeria (45). Another more recent and prominent example is medical populism in the United States during the COVID-19 pandemic. There have been reports of long standing distrust in the health care system in the United States, even suggestions for it to be classed as a public health issue given the potential impact on health outcomes (46). This distrust was mobilized during the pandemic by politicians, prominent personalities, and conspiracy theories in a manner that pit ‘the people’ against a grouping of ‘experts, elites, and public institutions’ [(47, 48), pp. 87–88, (14), pp. 221–222]. Notable examples include then President Trump drawing strong associations between the state of the economy and the impact of public health interventions in general or framing particular control measures like masking as an infringement of personal choice and individual freedom [(49), p. 1423]. Research shows that disparaging characterization of public health institutions and their handling of the pandemic was among the strongest indicators negatively influencing the decision to vaccinate [(50), p. 93].

The examples raised in this perspective paper highlight cases where engaging distrust during crises contributed to achieving public health goals along with cautionary cases where accomplishing population health outcomes became difficult. These examples, however, are particular instances and further research is required to validate the overarching claim: during crisis periods trust can be built indirectly and more effectively by engaging with existing distrust in a manner that acknowledges the ways in which public needs and desires have not been met. Partnering with already trusted actors is an important means to effectively engage with distrust around public health interventions. This perspective paper also raises the point that if public distrust and existing health inequity are not addressed, then space is created during crisis-periods for distrust to be mobilized in ways that compromise achieving public health outcomes.

## References

[1] Whyte KP Crease RP. Trust, expertise, and the philosophy of science. Synthese. (2010) 177:411–25. doi: 10.1007/s11229-010-9786-3

[2] Luhmann N (2017). Trust and Power. Translated by Howard Davis, John Raffan, and Kathryn Rooney. Chichester West Sussex: Wiley.

[3] Kroeger F. The development, escalation and collapse of system trust: from the financial crisis to Society at Large. Eur Manag J. (2015) 33:431–7. doi: 10.1016/j.emj.2015.08.001

[4] Larson HJ Clarke RM Jarrett C Eckersberger E Levine Z Schulz WS. Measuring Trust in Vaccination: a systematic review. Hum Vaccin Immunother. (2018) 14:1599–609. doi: 10.1080/21645515.2018.1459252, PMID: 29617183 PMC6067893

[5] Bildtgård T. Trust in Food in modern and late-modern societies. Soc Sci Inf. (2008) 47:99–128. doi: 10.1177/0539018407085751

[6] Shapin S. A social history of truth: Civility and science in seventeenth-century England. Chicago: University of Chicago Press (2011). 11620329

[7] Baier A. (1997). “Trust and antitrust.” In Feminist social thought: A reader, edited Meyers Diana T., 605–629. New York: Routledge.

[8] Lewicki RJ McAllister DJ Bies RJ. Trust and distrust: new relationships and realities. Acad Manag Rev. (1998) 23:438–58. doi: 10.2307/259288

[9] Boin A McConnell A Hart P‘T. Governing the pandemic: The politics of navigating a mega-crisis. Cham: Springer International Publishing (2021).

[10] Kattumana T Heyerdahl LW Nguyen TT Dielen S Grietens KP Vandamme A-M. More than one crisis: COVID-19 response actors navigating multi-dimensional crises in Flanders, Belgium. Crit Public Health. (2023) 33:566–78. doi: 10.1080/09581596.2023.2240480

[11] Janzwood S Homer-Dixon T. (2022). “What is a global Polycrisis?” 4. Cascade Institute. Available online at: https://cascadeinstitute.org/technical-paper/what-is-a-global-polycrisis/ (Accessed August 24, 2024).

[12] Carel H. The locked-down body: embodiment in the age of pandemic. Philosopher. (2020):12–7.

[13] Froese T Broome M Carel H Humpston C Malpass A Mori T. The pandemic experience: a Corpus of subjective reports on life during the first wave of COVID-19 in the UK, Japan, and Mexico. Front Public Health. (2021) 9:725506. doi: 10.3389/fpubh.2021.725506, PMID: 34490201 PMC8418530

[14] Kattumana T Byrne T. On dissent against public health interventions: a phenomenological perspective during the COVID-19 pandemic In: Byrne T Wenning M, editors. The right to resist: Philosophies of dissent. London: Bloomsbury (2023). 207–33.

[15] Dewar C Keller S Malhotra V. (2024). The mindsets of a leader: CEO Excellence book interview. Interview by Raju Narisetti. Available online at: https://www.mckinsey.com/featured-insights/mckinsey-on-books/impacting-audiences-across-the-globe-ceo-excellence-revisited (Accessed January 16, 2025).

[16] Gramlich J Funk C. (2020). “Black Americans face higher COVID-19 risks, are more hesitant to trust medical scientists, get vaccinated.” *Pew Research Center* (blog). Available online at: https://www.pewresearch.org/fact-tank/2020/06/04/black-americans-face-higher-covid-19-risks-are-more-hesitant-to-trust-medical-scientists-get-vaccinated/ (Accessed January 4, 2025).

[17] Hamel L Lopes L Muñana C Samantha A Brodie M. (2020). “KFF/the undefeated survey on race and health.” *KFF* (blog). Available online at: https://www.kff.org/report-section/kff-the-undefeated-survey-on-race-and-health-main-findings/ (Accessed January 4, 2025).

[18] Kattumana T. Trust, vaccine hesitancy, and the COVID-19 pandemic: a phenomenological perspective. Soc Epistemol. (2022) 36:641–55. doi: 10.1080/02691728.2022.2115325

[19] Bunch L. A tale of two crises: addressing Covid-19 vaccine hesitancy as promoting racial justice. HEC Forum. (2021) 33:143–54. doi: 10.1007/s10730-021-09440-0, PMID: 33464452 PMC7814857

[20] Best AL Fletcher FE Kadono M Warren RC. Institutional distrust among African Americans and building trustworthiness in the COVID-19 response: implications for ethical public health practice. J Health Care Poor Underserved. (2021) 32:90–8. doi: 10.1353/hpu.2021.0010, PMID: 33678683 PMC7988507

[21] Eligon J (2020). “Black doctor dies of Covid-19 after complaining of racist treatment.” The New York Times. Available online at: https://www.nytimes.com/2020/12/23/us/susan-moore-black-doctor-indiana.html (Accessed January 15, 2025).

[22] Hoffman KM Trawalter S Axt JR Norman Oliver M. Racial Bias in pain assessment and treatment recommendations, and false beliefs about biological differences between blacks and whites. Proc Natl Acad Sci. (2016) 113:4296–301. doi: 10.1073/pnas.1516047113, PMID: 27044069 PMC4843483

[23] Givens R. One of us. N Engl J Med. (2021) 384:e18. doi: 10.1056/NEJMpv2100228, PMID: 33503341

[24] Gamble VN. Under the shadow of Tuskegee: African Americans and health care. Am J Public Health. (1997) 87:1773–8. doi: 10.2105/AJPH.87.11.1773, PMID: 9366634 PMC1381160

[25] Brandon DT Isaac LA LaVeist TA. The legacy of Tuskegee and Trust in Medical Care: is Tuskegee responsible for race differences in mistrust of medical care? J Natl Med Assoc. (2005) 97:951–6. PMID: 16080664 PMC2569322

[26] Oladele CR McKinney TL Tolliver D Tuckson R Dawes D Nunez-Smith M. (2022). “The state of black American and COVID-19: A two year Assesment.” The Black Coalition Against COVID. Available online at: https://blackcoalitionagainstcovid.org/the-state-of-black-america-and-covid-19/ (Accessed January 13, 2025).

[27] Padamsee TJ Bond RM Dixon GN Hovick SR Na K Nisbet EC. Changes in COVID-19 vaccine hesitancy among black and white individuals in the US. JAMA Netw Open. (2022) 5:e2144470. doi: 10.1001/jamanetworkopen.2021.44470, PMID: 35061038 PMC8783270

[28] Howard University. Making it plain… African-Americans and the COVID-19 vaccine, PART 1. Washington DC: Howard University (2020).

[29] Bulik BS (2021). “Black doctors read COVID tweets in fun, fact-filled campaign to raise vaccination awareness.” Fierce pharma. Available online at: https://www.fiercepharma.com/marketing/black-doctors-read-covid-tweets-fun-fact-filled-campaign-to-raise-vaccination-awareness (Accessed January 13, 2025).

[30] Macaya HR Renton A Wilkinson P Wagner M Hayes M Melissa. (2020). “Access to Covid-19 testing is part of why black Americans face higher infection rates, expert says.” CNN 2020. Available online at: https://www.cnn.com/world/live-news/coronavirus-pandemic-06-18-20-intl/index.html (Accessed January 13, 2025).

[31] Dada D Djiometio JN McFadden SAM Demeke J Vlahov D Wilton L. Strategies that promote equity in COVID-19 vaccine uptake for black communities: a review. J Urban Health. (2022) 99:15–27. doi: 10.1007/s11524-021-00594-3, PMID: 35018612 PMC8751469

[32] Meher B Acharya AK. De-identifying the distressed in the transgender community related to their identity formation and discrimination in India. Genealogy. (2022) 6:1–12. doi: 10.3390/genealogy6040092

[33] United Nations Development Programme. (2010). “Hijras/transgender women in India: HIV, human rights and social exclusion.” Issue brief. India. Available online at: https://archive.nyu.edu/bitstream/2451/33612/2/hijras_transgender_in_india.pdf (Accessed February 12, 2024).

[34] Thankachan A Rathore P Kumar S Shweta VK Haokip N Bhatnagar S. Challenging concerns of transgender community amidst COVID-19. Indian J Palliat Care. (2020) 26:S166–7. doi: 10.4103/IJPC.IJPC_166_20, PMID: 33088112 PMC7534990

[35] Chakrapani V Babu P Ebenezer T. Hijras in sex work face discrimination in the Indian health-care system. Res Sex Work. (2004):12–4.

[36] Acharya AK Clark JB Behera SS. COVID-19 pandemic and transgender migrant women in India: socio-economic vulnerability and vaccine hesitancy. J Migrat Health. (2023) 8:100204. doi: 10.1016/j.jmh.2023.100204, PMID: 38028887 PMC10654218

[37] Choudhary P. (2021). “India’s COVID-19 vaccination drive is failing the transgender community.” *Fair Observer* (blog). Available online at: https://www.fairobserver.com/region/central_south_asia/preeti-choudhary-transgender-rights-covid-19-vaccination-india-news-15521/ (Accessed January 17, 2025).

[38] Soni GK Seth S Arora S Singh K Kumari A Kanagat N. Harnessing the power of collaboration to expand the coverage and equity of COVID-19 vaccinations in India: a community collaboration model. Vaccine. (2023) 11:1022. doi: 10.3390/vaccines11061022, PMID: 37376411 PMC10304198

[39] Choudhary P. (2021). “Less than 4% vaccinated – transgender Indians have been ‘Othered’ by vaccines and forms.” The Print. Available online at: https://theprint.in/opinion/less-than-4-vaccinated-transgender-indians-have-been-othered-by-vaccines-and-forms/659091/ (Accessed January 17, 2025).

[40] Swasti. Covid-19 playbook. Bangalore: Swasti (2021).

[41] Hamilton M. (2022). “Bringing Covid-19 vaccines to millions in India’s stigmatized communities.” *The Rockefeller Foundation* (blog). Available online at: https://www.rockefellerfoundation.org/grantee-impact-stories/bringing-covid-19-vaccines-to-millions-in-indias-stigmatized-communities/ (Accessed January 21, 2025).

[42] Lasco G Curato N. Medical Populism. Soc Sci Med. (2019) 221:1–8. doi: 10.1016/j.socscimed.2018.12.006, PMID: 30553118

[43] Lasco G Larson HJ. Medical populism and immunisation Programmes: illustrative examples and consequences for public health. Glob Public Health. (2020) 15:334–44. doi: 10.1080/17441692.2019.1680724, PMID: 31630625

[44] Raufu A. Polio vaccine plans may run into problems in Nigeria. BMJ. (2003) 327:380. doi: 10.1136/bmj.327.7410.380-c, PMID: 12907514 PMC1142510

[45] Ghinai I Willott C Dadari I Larson HJ. Listening to the rumors: what the northern Nigeria polio vaccine boycott can tell us ten years on. Glob Public Health. (2013) 8:1138–50. doi: 10.1080/17441692.2013.859720, PMID: 24294986 PMC4098042

[46] Armstrong K Rose A Peters N Long JA McMurphy S Shea JA. Distrust of the health care system and self-reported health in the United States. J Gen Intern Med. (2006) 21:292–7. doi: 10.1111/j.1525-1497.2006.00396.x, PMID: 16686803 PMC1484714

[47] Gugushvili A Koltai J Stuckler D McKee M. Votes, populism, and pandemics. Int J Public Health. (2020) 65:721–2. doi: 10.1007/s00038-020-01450-y, PMID: 32740685 PMC7394929

[48] Kattumana T. Alternative credibility, phenomenological empathy, and the Plandemic: Trust in Conspiracy Theories during the COVID-19 pandemic. J Digital Soc Res. (2023) 5:85–108. doi: 10.33621/jdsr.v5i3.146

[49] Lasco G. Medical populism and the COVID-19 pandemic. Glob Public Health. (2020) 15:1417–29. doi: 10.1080/17441692.2020.1807581, PMID: 32780635

[50] Sabahelzain MM Hartigan-Go K Larson HJ. The politics of Covid-19 vaccine confidence. Curr Opin Immunol. (2021) 71:92–6. doi: 10.1016/j.coi.2021.06.007, PMID: 34237648 PMC8206618

